# Molecular basis of a novel adaptation to hypoxic-hypercapnia in a strictly fossorial mole

**DOI:** 10.1186/1471-2148-10-214

**Published:** 2010-07-16

**Authors:** Kevin L Campbell, Jay F Storz, Anthony V Signore, Hideaki Moriyama, Kenneth C Catania, Alexander P Payson, Joseph Bonaventura, Jörg Stetefeld, Roy E Weber

**Affiliations:** 1 Department of Biological Sciences, University of Manitoba, Winnipeg, MB R3T 2N2 Canada; 2 School of Biological Sciences, University of Nebraska, Lincoln, NE 68588, USA; 3 Department of Biological Sciences, Vanderbilt University, Nashville, TN 37235, USA; 4 Duke University Medical Center and Nicholas School of the Environment Marine Laboratory, Beaufort, NC 28516, USA; 5 Department of Chemistry, University of Manitoba, Winnipeg, MB R3T 2N2 Canada; 6 Zoophysiology, Institute of Biological Sciences, University of Aarhus, Aarhus, DK 8000, Denmark

## Abstract

**Background:**

Elevated blood O_2 _affinity enhances survival at low O_2 _pressures, and is perhaps the best known and most broadly accepted evolutionary adjustment of terrestrial vertebrates to environmental hypoxia. This phenotype arises by increasing the intrinsic O_2 _affinity of the hemoglobin (Hb) molecule, by decreasing the intracellular concentration of allosteric effectors (e.g., 2,3-diphosphoglycerate; DPG), or by suppressing the sensitivity of Hb to these physiological cofactors.

**Results:**

Here we report that strictly fossorial eastern moles (*Scalopus aquaticus*) have evolved a low O_2 _affinity, DPG-insensitive Hb - contrary to expectations for a mammalian species that is adapted to the chronic hypoxia and hypercapnia of subterranean burrow systems. Molecular modelling indicates that this functional shift is principally attributable to a single charge altering amino acid substitution in the β-type δ-globin chain (δ136Gly→Glu) of this species that perturbs electrostatic interactions between the dimer subunits via formation of an intra-chain salt-bridge with δ82Lys. However, this replacement also abolishes key binding sites for the red blood cell effectors Cl^-^, lactate and DPG (the latter of which is virtually absent from the red cells of this species) at δ82Lys, thereby markedly reducing competition for carbamate formation (CO_2 _binding) at the δ-chain N-termini.

**Conclusions:**

We propose this Hb phenotype illustrates a novel mechanism for adaptively elevating the CO_2 _carrying capacity of eastern mole blood during burst tunnelling activities associated with subterranean habitation.

## Background

**Figure 1:**
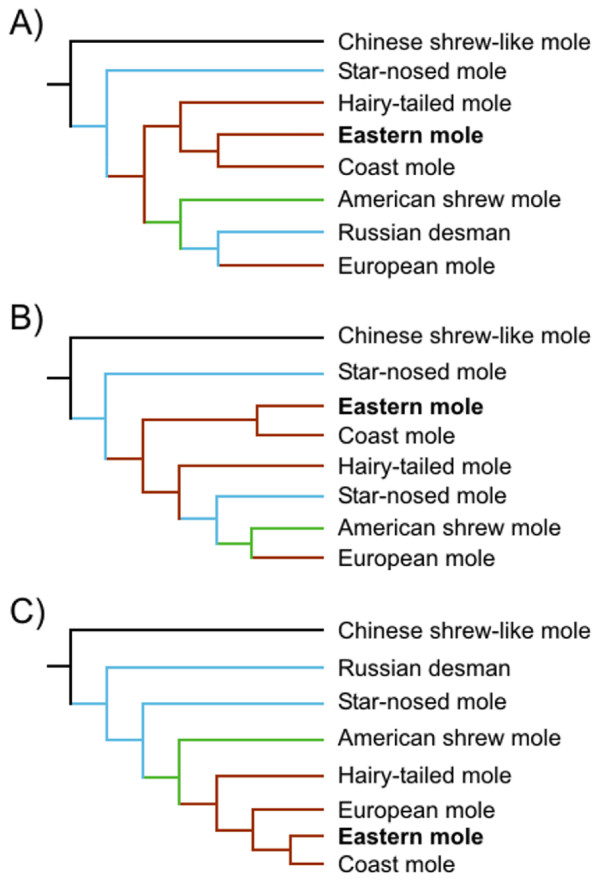
**Phylogenetic hypothesis of the Family Talpidae**. (A) Shinohara et al. 2003 [6], (B) Motokawa 2004 [7], and (C) Whidden 2000 [8]. Line colours refer to the habitats exploited by each: high-alpine terrestrial (black), semi-aquatic (blue), fossorial (brown) and semi-fossorial (green). Note that all topologies suggest that the lineage leading towards present-day eastern moles arose following an extensive period of fossorial evolution.

Among mammals that are adapted to hypoxic environments, only subterranean species are also obliged to breathe air with elevated concentrations of carbon dioxide [1]. Within this select group, the 25 or so species of fossorial moles (Family Talpidae) are among the few that live exclusively underground. These small (70-130 g) insectivores favour moist, invertebrate-rich substrates to excavate extensive closed-burrow systems. Due to the impeded gas exchange of damp soils with surface air, these animals are chronically exposed to hypoxic and hypercapnic environments (14.3% O_2 _and 5.5% CO_2 _have been recorded within mole tunnels; [2]). The high metabolic costs of burrowing, in terms of O_2 _consumption and CO_2 _production, are exacerbated by the obligate re-breathing of expired air while tunnelling, and may therefore require adaptive modifications in hemoglobin (Hb) function. Not surprisingly, whole blood O_2 _affinity of the European mole, *Talpa europaea*, is much higher (its half saturation O_2 _pressure or P_50 _is 10-15 mm Hg lower) than those of terrestrial mammals of similar size [1,3,4]. This feature has been attributed to a reduced affinity of European mole Hb for 2,3-diphosphoglycerate (DPG) [4,5], which binds to a cluster of positively charged residues between the β-type chains and stabilizes the low affinity (deoxy) conformation of the molecule. However, it is not known whether other fossorial members of the family Talpidae possess similar specializations to the subterranean environment. This question is of interest in light of recent evidence which suggests that New World (Scalopini) and Old World (Talpini) moles have convergently invaded the subterranean habitat [[6,7], but see [8]; Figure 1].

In order to assess the functional and evolutionary adaptations of mole Hbs, and their mechanistic basis, we determined the coding sequences of the adult-expressed α-and β-type globin chains of coast mole (*Scapanus orarius*) and eastern mole (*Scalopus aquaticus*), two closely related, strictly fossorial species that are endemic to Western and Eastern North America, respectively, and we investigated the ligand-binding properties of their Hb components. Based on unexpected findings for *S. aquaticus *Hb, we also measured oxygen-binding properties of whole blood for both species and the semi-aquatic star-nosed mole (*Condylura cristata*). To gauge whether the distinctive oxygenation properties of eastern mole Hb are accompanied by complementary or compensatory physiological adjustments, we also measured hematological and muscle biochemical properties of the two fossorial species.

## Results

### Hb-O_2 _binding properties

**Figure 2:**
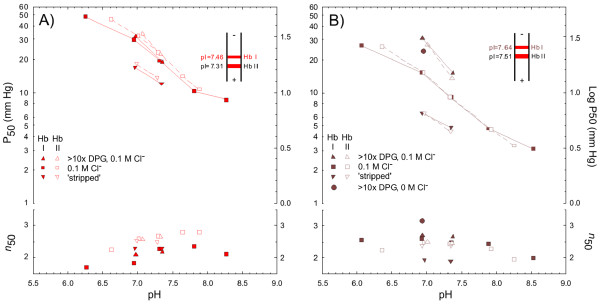
**Values of PO_2 _and Hill's cooperativity coefficient at half oxygen saturation (P_50 _and *n*_50_) for the two major isohemoglobin components of (A) eastern and (B) coast moles at 37°C, and their pH dependence in the absence and presence of added Cl^- ^(0.1 M) and 2,3-DPG (DPG/Hb_4 _ratio > 50)**. *Upper right insets: *diagrams of isoelectric focusing column at the end of focusing illustrating the relative abundance of the individual hemoglobin components and their isoelectric points at 5°C.

Both gel electrophoresis and isoelectric focusing (IEF; inset of Figure 2) revealed the presence of two major isoHb components (the tetrameric Hbs of each component possess distinct α-type globin chains; see below) in all individuals examined (*n *= 3 coast moles; *n *= 4 eastern moles), where the Hb I:Hb II ratio approximated 60:40 and 35:65, respectively. In addition to these major components, one of the eastern mole specimens possessed a minor cathodic Hb component that represented ~5% of the hemolysate and exhibited oxygen-binding properties similar to those of the major components (data not shown). Preparative IEF revealed relatively high isoelectric points (the pH at which the protein carries a net neutral charge) for the CO-bound Hb components of both species at 5°C (inset of Figure 2).

Oxygen equilibration curves revealed striking functional differences between the Hbs of the two species (Figure 2), with 'stripped' (cofactor-free) Hbs of the eastern mole exhibiting P_50_'s (13.8 and 14.8 mm Hg for Hbs I and Hb II, respectively, at 37°C, pH 7.2) that are nearly three-fold higher than those of the coast mole (P_50 _= 5.3 and 5.1 mm Hg, respectively). Eastern mole Hbs also exhibited slightly lower chloride sensitivities (Δlog P_50_/Δlog [Cl^-^]; Table 1), though the Hbs of both species showed similar cooperativity coefficients (Figure 2). Significantly, the O_2_-affinity of coast mole Hbs was sharply reduced in the presence of saturating concentrations of DPG, a trait not observed in eastern mole Hbs (Table 1, Figure 2). Even under saturating DPG concentrations the P_50 _of coast mole Hbs (18.3-19.7 mm Hg at pH 7.2) remained lower than those of eastern mole Hbs in the absence of this organophosphate (22.2-26.0 mm Hg; Figure 2). Similarly, oxygenation enthalpies (Δ*H*) of coast mole Hbs at pH 7.2 and in 0.1 M Cl^- ^media (-7.6 to -9.7 kJ mol^-1 ^O_2_; Table 1) were lower than those of eastern moles (-10.3 to -13.7 kJ mol^-1^), though both were low compared to human Hb A at pH 7.4 (-41.0 kJ mol^-1^; [9]).

**Table 1:** Heterotropic effects at half-saturation (P_50_) for coast and eastern mole hemoglobin

	**Eastern mole**		**Coast mole**	
		
	**Hb I**	**Hb II**	**Hb I**	**Hb II**
		
Δlog P_50_/Δlog [Cl^-^]^1,2^	0.21	0.25	0.31	0.33
Δlog P_50_± 2.5 mM DPG^1,3^	0.02	0.02	0.26	0.23
				
Δlog P_50_/ΔpH^4^				
25°C, 0.1 M Cl^-^	-0.57	-0.61	-0.55	-0.62
37°C, 'stripped'	-0.40	-0.41	-0.32	-0.44
37°C, 0.1 M Cl^-^	-0.51	-0.52	-0.54	-0.59
37°C, 0.1 M Cl^- ^+ 2.5 mM DPG	-0.63	-0.73	-0.86	-0.78
				
Oxygenation enthalpy; Δ*H *(kJ mol^-1 ^O_2_)^5^	-13.7	-10.3	-9.7	-7.6

^1^pH = 7.2

^2^[DPG] = 0 mM

^3^[Cl^-^] = 0.1 M

^4^over pH range 6.9-7.4; PCO_2 _= 0 mm Hg

^5^[Cl^-^] = 0.1 M; [DPG] = 0 mM; pH = 7.2

### O_2_-binding properties of whole blood

**Figure 3:**
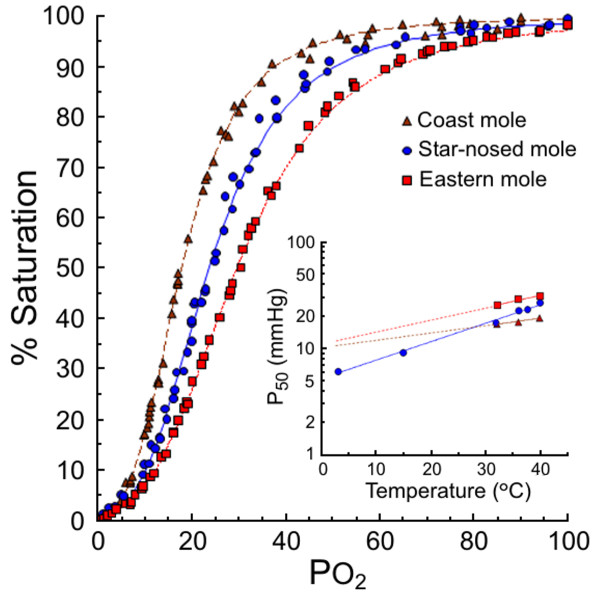
**Oxygen equilibration curves of freshly drawn coast (■) eastern (▲) and star-nosed mole (●) blood at 36°C and a PCO_2 _of 38 mm Hg**. *Inset: *The effect of temperature on the half-saturation pressure (P_50_) of whole blood of these three species.

In accordance with the observed differences in the oxygen affinity of coast vs. eastern mole Hb components in the presence of allosteric effectors (Figure 2), the P_50 _of freshly drawn coast mole blood (17.7 mm Hg at 36°C) was substantially lower than that from the eastern mole (28.8 mm Hg), while that of the amphibious star-nosed mole was intermediate (22.5 mm Hg; Figure 3, Table 2). However, whole blood pH was notably lower in eastern mole (pH = 7.38) than in the coast and star-nosed moles (7.60 and 7.55, respectively). Even when corrected to pH 7.4, a clear difference in whole-blood O_2_-affinity of these two fossorial species was evident (P_50 _= 21.9 vs. 26.9 mm Hg, respectively). The CO_2 _Bohr coefficients (determined by measurements whereby the pH is changed by varying PCO_2_) of blood from coast mole (-0.52) and star-nosed mole (-0.40) were within the typical mammalian range (-0.39 to -0.62; [10]), while that of the eastern mole was unusually high (-0.78; Table 2). Consistent with our Hb data, the blood P_50 _values of both fossorial species showed low temperature sensitivities, with the Hb-oxygenation reaction being less exothermic in coast mole (Δ*H*=-1.0 kJ mol^-1 ^O_2_; Table 2) than in the eastern mole (-8.3 kJ mol^-1^). Surprisingly, blood-O_2 _affinity of the semi-aquatic star-nosed mole was more strongly governed by temperature (inset of Figure 3), as reflected by its high oxygenation enthalpy (-29.9 kJ mol^-1 ^O_2_; Table 2) relative to the fossorial mole species.

**Table 2:** Respiratory characteristics of eastern, coast and star-nosed mole whole blood

	**36°C**				**36°C**				**ΔlogP**_**50/ΔpH**_^4^	**32°C**		**40°C**		**Δ*H***^**5**^**(kJ mol**^**-1**^**O**_**2**_)	**Δlog P**_**50**_**/ΔT**
	**PCO**_**2 **_**= 38 mm Hg**				**PCO**_**2 **_**= 38 mm Hg**					**PCO**_**2 **_**= 38 mm Hg**		**PCO**_**2 **_**= 38 mm Hg**			
							
	**pH**	**P**_**50**_^**1**^	**P**_**d**_^**2**^	***n***_**50**_^**3**^	**pH**	**P**_**50**_	**P**_**d**_	***n***_**50**_		**P**_**50**_	***n***_**50**_	**P**_**50**_	***n***_**50**_		
						
Eastern mole	7.36	28.8	21.9	2.76	7.76	14.1	10.1	2.54	-0.78	25.3	2.78	31.0	2.71	-8.3	0.011
Coast mole	7.58	17.7	13.6	2.84	8.48	6.1	4.2	2.44	-0.52	16.9	2.85	19.3	2.82	-1.0	0.008
Star-nosed mole	7.56	22.5	17.1	2.76	8.61	8.0	6.0	2.86	-0.40	17.3	2.95	26.6	2.90	-29.9	0.023

^1^P_50 _(mm Hg) = PO_2 _corresponding to 50% oxygen saturation

^2^P_d _(mm Hg) = P50 × ((*n*_50_-1)/(*n*_50_+1))^1/*n50*^; represents arterial PO_2 _at which oxygen offloading is maximal [41]

^3^*n*_50 _= Hill coefficient; slope of log (%oxyHb/%deoxyHb) versus log PO_2_

^4^CO_2_-Bohr effect; pH varies by CO_2 _titration

^5^Overall heat of oxygenation.

Prominent differences were also detected in specific hematological parameters of the two fossorial species, with the most significant being the > 15 fold lower DPG levels in the erythrocytes of eastern mole relative to coast mole (Table 3). Tissue myoglobin concentration was also consistently lower in eastern moles (by 10-20%), although this difference was only significant for hindlimb muscles. Conversely, hematocrit and Hb concentrations were 20.5% and 10.3% higher, respectively, in eastern mole blood than in coast mole blood (Table 3).

**Table 3:** Hematological parameters, skeletal muscle myoglobin concentrations and buffering capacities (± 1 SE) of coast and eastern moles (samples sizes are indicated in brackets)

Variable	Eastern mole	Coast mole
Hematocrit (%)	56.4 ± 1.3 (7)^†^	46.8 ± 2.0 (11)^1^
Hemoglobin (g dL^-1^)	19.2 ± 0.7 (7)	17.4 ± 0.8 (11)^1^
RBC (10^6^/mm^3^)	12.58 ± 0.32 (3)	10.48 (1)
MCV (μ^3^)	46.0 ± 1.3 (3)	42.6 (1)
MCH (pg)	15.3 ± 0.6 (3)	13.5 (1)
MCHC (g L^-1^)	333 ± 7 (3)	318 (1)
2,3-DPG (mM L^-1 ^RBC)	0.45 ± 0.06 (4)^†^	7.09 ± 0.20 (3)
Myoglobin content (mg g^-1 ^wet tissue)		
Heart	8.34 ± 0.38 (6)	9.24 ± 0.28 (7)^1^
Forelimb	10.98 ± 0.64 (6)	12.10 ± 0.25 (10)^1^
Hindlimb	8.56 ± 0.21 (6)^†^	10.61 ± 0.56 (10)^1^
Buffering capacity, β (Slykes)*		
Forelimb	43.86 ± 3.29 (6)	37.33 ± 2.10 (10)^1^
Hindlimb	44.99 ± 5.54 (6)	38.94 ± 1.77 (10)^1^
Plasma pH (pH*e*)	7.42 ± 0.01 (6)	---
Intraerythrocytic pH (pH*i*)	7.21 ± 0.00 (6)	---
Plasma osmolarity (mOsm)	333 ± 2 (6)	---

^†^Interspecific differences are significant (*P *< 0.05)

*1 Slyke = μmoles of base required to titrate the pH of 1 g of wet muscle by 1 pH unit

^1^data from [45]

### Identification of Hb isoforms

**Figure 4:**
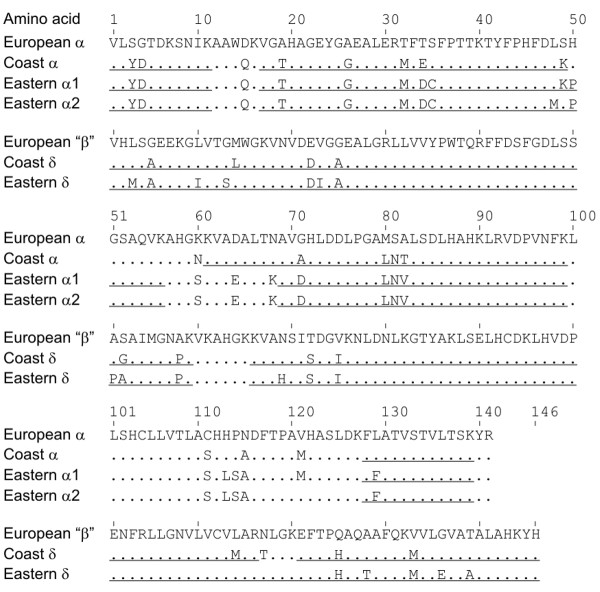
**Alignment of mole α- and δ-globin chains**. Amino acid residues of coast and eastern moles were deduced from cDNA nucleotide sequences and are shown only at positions where they differ from those of European moles [5]. The underlined portions of each amino acid sequence denote the coverage of matched peptides in the mass spectrometry analysis (see text for details).

One β-like and two α-like globin cDNAs were obtained from three separate eastern moles, while single α- and β-like globin cDNAs were obtained from three coast moles examined (Figure 4). A mass spectrometic analysis of eastern mole Hb components revealed the presence of two distinct α-globin sequences that match the two known *HBA *cDNA sequences ('α1' and 'α2'; Figure 4). The analysis revealed the presence of a single β-like globin sequence that matched the eastern mole *HBD *(δ) cDNA sequence (see below). These results confirm that the two Hb isoforms of eastern mole have different α-chain subunits that are distinguished by three amino acid substitutions: 48Leu→Met, 49Lys→Ser, and 121Met→Val (α1→α2 in each case). The mass spectrometry analysis of coast mole Hb also revealed highly significant matches to coast mole cDNA sequences. However, since the analysis revealed no evidence for structurally distinct Hb isoforms, the distinct bands in the isoelectric focusing gels likely reflect some form of *in vivo *post-translational modification. The electrophoretic mobility patterns of the two fractions (inset of Figure 2B) are consistent with a deamidation reaction, whereby a portion of the amide side-chain moieties of specific Asn or Gln residues of Hb are converted to carboxyl groups [11,12]. Based on the primary sequences of the coast mole globin chains (Figure 4), the most probable scenario is deamidation of α60Asn→Asp, which is adjacent to two residues (α58His and α59Gly) that are often associated with this non-enzymatic reaction [11,12].

### Phylogenetic analyses of mole β-like genes

Phylogenetic surveys of the β-globin gene family have revealed that, in most mammalian species, the β-type globin chains of adult Hb are encoded by one or more copies of the *HBB *(β) gene [13,14]. However, in the eulipotyphlan (moles, shrews, hedgehogs and solenodons) species that have been examined to date (Eurasian shrew, *Sorex araneus*, and African pygmy hedgehog, *Atelerix albiventris*) the β-type globin chains of adult Hb are encoded by multiple copies of the paralogous *HBD *(δ) gene [13]. Because recent molecular phylogenies now suggest that *Sorex *and *Atelerix *are sister taxa and are closely related to moles [15], it is still unknown whether *HBD *supplanted the *HBB *gene before or after the shrew/hedgehog common ancestor diverged from the stem lineage of talpid moles.

**Figure 5:**
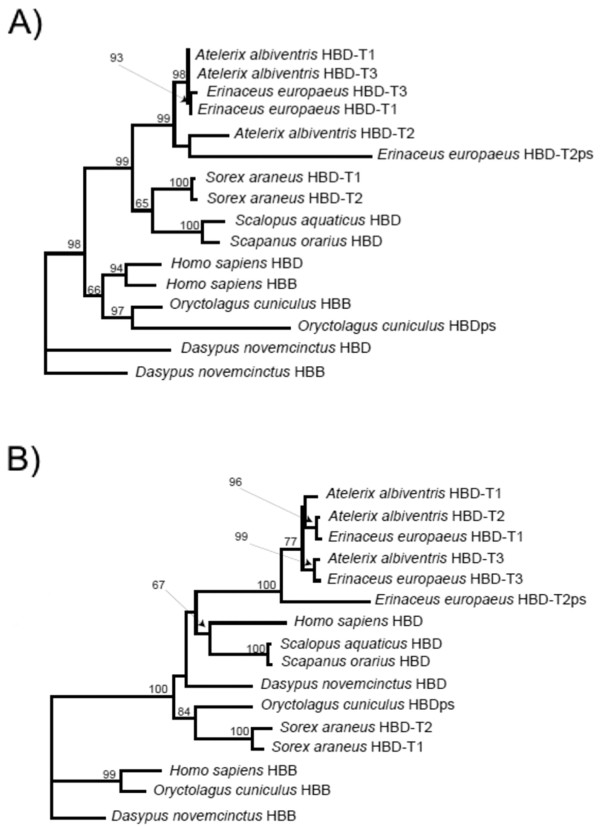
**Phylogenetic analysis of mole β-like globin genes**. Maximum likelihood phylograms depicting relationships among mammalian β-like globin genes based on (A) 477 bp of coding sequence, and (B) 938 bp of intron 2. With the exception of the sequences from eastern mole (*Scalopus aquaticus*) and coast mole (*Scapanus orarius*), all β-like globin sequences were retrieved from full genome assemblies (see text for details). Both trees demonstrate that the β-like globin genes of *Scalopus *and *Scapanus *are orthologous to the *HBD *genes of other mammals. Because mammals typically possess multiple tandemly duplicated copies of adult beta-like globin genes, we index each paralog with the symbol -T followed by a number that corresponds to the 5' to '3' linkage order. Pseudogene sequences are indicated by the suffix 'ps'.

We used phylogenetic reconstructions of coding sequences and intron 2 sequences to determine whether the β-like globin genes of eastern mole and coast mole are orthologous to the *HBB *or *HBD *genes of other eutherian mammals. Phylogenetic reconstructions based on coding sequence indicated that orthologous relationships may be partly obscured by a history of concerted evolution, mediated by unequal crossing-over or interparalog gene conversion (a form of nonreciprocal recombination between duplicated genes) [13]. For example, a history of gene conversion is indicated by the fact that the human *HBB *gene is more similar to the human *HBD *gene than it is to the orthologous *HBB *gene in rabbit or armadillo (Figure 5A). By contrast, the phylogeny based on intron 2 sequence shows that the β-like globin genes of all eulipotyphlan species examined - together with the *HBD *genes of human, rabbit, and armadillo - form a well-supported monophyletic group with high bootstrap support (Figure 5B). This set of relationships indicates that the adult-expressed β-like globin genes of moles are orthologous to the *HBD *genes of other eutherian mammals. Thus, the *HBD *gene appears to have supplanted the *HBB *gene in the common ancestor of shrews, hedgehogs and moles.

## Discussion

The pioneering work of Bunn [16] demonstrated that mammalian Hbs fall into two discrete categories based on their ligand-binding properties. Most species have Hbs with intrinsically high O_2 _affinity that is markedly reduced in the red cell by physiological concentrations of DPG (4-10 mM l^-1 ^RBC), which stabilizes the tense (deoxy) state of Hb by electrostatic binding within a cationic pocket (formed by β1Val, β2His, β82Lys and β143His) between the β-chains [17]. Conversely, the Hbs of feloids, ruminants and two species of lemur exhibit low O_2 _affinities and respond only weakly to organophosphates [17], which moreover occur in low concentrations in the red cells [16]. The β-type chains of these animals share two features without exception: the substitution of a hydrophilic residue with a hydrophobic residue at β2 [18], and the presence of β5Ala. The latter residue is thought to give the first turn of helix A more flexibility [19], allowing the modified N-termini to be drawn closer to the hydrophobic cavity between the β chains and thus mimicking DPG binding [18].

Though δ5Ala is present in eastern (and coast) moles, contrary to our expectations, all known phosphate-binding sites are conserved in the β-type δ-chains of moles (Figure 4). Significantly, however, eastern moles possess a novel δ136Gly→Glu replacement that introduces two anionic residues into the positively charged central cavity of the Hb tetramer. This position is essentially invariant among mammals. However, a rare Hb mutant in humans, Hb Hope, is characterized by a comparable charge altering replacement (β136Gly→Asp) and, interestingly, exhibits functional properties similar to that of eastern mole Hb [20,21]. It has been proposed that the altered behaviour of Hb Hope arises from the formation of an intra-chain salt bridge between the carboxyl (COO^-^) group of β136Asp and the charged α-amino group (NH_2_^+^) of β1Val [22], thus deleting a pair of DPG binding sites and stabilizing the T-state molecule via the newly established ionic linkage [21,23,24]. However, the electrophoretic properties of Hb Hope remain unchanged at pH 8.6 [25], where the N-terminius is expected to be deprotonated (its pKa is ~6.4-6.8; [26,27]) and the β1Val-β136Asp salt-bridge thus destabilized. Additionally, given that this proposed interaction deletes a maximum of two (of seven) docking sites for DPG, the near complete abolishment of organophosphate binding to Hb Hope is difficult to explain. Indeed, Hb variants that lack DPG binding sites at the N-terminus (e.g., Hb Raleigh β1Val→Ac-Ala; [28]), at β2 (e.g., Hb Fukuoka β2His→Tyr, Hb Okayama His→Gln; [29]) and at β143 (e.g., Hb Little Rock, β143His→Gln; [30]) only exhibit modest reductions in DPG sensitivity. These inconsistencies suggest another molecular mechanism may underlie the drastic functional changes found in Hb Hope and the eastern mole protein.

**Figure 6:**
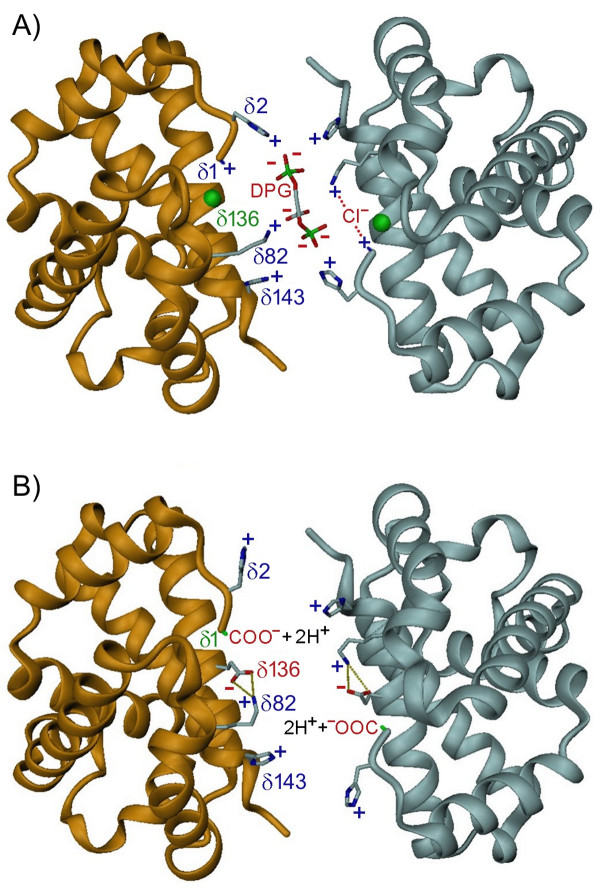
**Molecular models of the central cavity between the two δ-globin chains of (A) coast and (B) eastern mole hemoglobins**. In coast mole deoxyhemoglobin, this cavity is lined by 8 cationic residues (δ1Val, δ2His, δ82Lys and δ143His) that form competitive binding sites for anions (Cl^-^, lactate and DPG) that stabilize the low O_2 _affinity T-state conformation. In eastern mole deoxyhemoglobin, the carboxylate side chain of δ136Glu forms a salt bridge with ε-amino group of δ82Lys of the same chain, thus deleting key docking sites for these anions, and reducing competition for CO_2 _binding (carbamate formation) at the N-terminus (δ1Val). This change is expected to markedly increase the maximal CO_2 _carrying capacity of eastern mole blood.

In accord with this suggestion, our structural model illustrates that the carboxyl side chain of δ136Glu forms a stable salt bridge with the nearby ε-amino group of δ82Lys (Figure 6). This association is consistent with the observed low electrophoretic mobility of Hb Hope at pH 8.6 [25], as the high pKa of the lysine side chain (~10.5) would stabilize the strong β136Asp-β82Lys bond over a wide pH range. Importantly, the δ136Gly→Glu replacement in eastern mole Hb (and the β136Gly→Asp change in Hb Hope) is also expected to neutralize the strong cationic charge of the δ82 lysyl side chain (which normally projects directly into the central cavity). This substitution should reduce electrostatic repulsion between the dimer subunits [19,31], thereby reducing the intrinsic O_2 _affinity of the R-state protein. Consistent with this expectation, the intrinsic O_2 _affinity of eastern mole Hb is ~2.8-fold lower than that of coast mole Hb in the physiological pH range (Figure 2). Finally, the strongly suppressed DPG sensitivities of human Hbs with residue replacements at β82 (e.g., Hb Rahere β82Lys→Thr and Hb Providence β82Lys→Asn/Asp; [32,33]) are qualitatively similar in magnitude to that of eastern mole Hb (Figure 2, Table 1). In this regard, it is notable that eastern moles also possess an unusual δ3Leu→Met substitution in the N-terminal region of the δ-chains (Figure 4). Aided by the increased flexibility provided by residues δ5Ala and δ130Phe [19], the longer side-chain of this residue (which points internally towards δ133Met) may promote an increased interaction of the β-type subunit A-helix with its hydrophobic core, and further inhibit DPG binding (an analogous residue change found in human fetal Hb, γ3Leu→Phe, is implicated in reducing the affinity of this respiratory protein for O_2 _and DPG [34]). Finally, the eastern mole δ-globin chains possess an additional, potentially significant replacement that introduces a polar hydroxyl group (δ128Ala→Thr) into the α_1_δ_1 _interface. This site is typically invariant in mammalian Hbs [22,35] and may be responsible for the larger P_50 _difference between cofactor-free Hbs of eastern mole and coast mole (2.6-2.9 fold at 37°C; Figure 2) than the 1.8-fold change caused by the β136Gly→Asp substitution of Hb Hope at this same temperature [20].

The chloride sensitivities of both major Hb components of the eastern mole (0.21-0.25) are about 30% less than those of coast mole Hbs (Table 1). Hb Hope also exhibits a markedly lowered Cl^- ^effect relative to Hb A [21], providing additional support for the view that this characteristic arises from the loss of two anion binding sites in the eastern mole protein (i.e., between δ1Val and δ82Lys of each dimer; see Figure 6A). However, it is of note that the P_50 _of coast mole Hb I exposed to saturating DPG in 0.1 M Cl^- ^media was higher than values obtained when either of these anions were present alone (Figure 3). This observation suggests that mole Hbs possess an extra chloride-binding site (with respect to human Hb A) that does not overlap with the DPG binding site. This inference is moreover consistent with the numerically low oxygenation enthalpies (see below) of both mole Hbs in 0.1 M Cl^- ^media (Table 1).

The 7.3 kJ mol^-1 ^O_2 _difference in overall oxygenation enthalpy of eastern mole whole blood relative to coast mole blood (Table 2) aligns well with the reduced exothermic contributions conferred by both the loss of Cl^- ^binding sites between δ1Val and δ82Lys (-3.4 kJ mol^-1 ^O_2_; Table 1), and by a lack of DPG binding (-3.1 kJ mol^-1 ^O_2_; [36]) to eastern mole Hb. Interestingly, the numerical Δ*H *values for whole blood (-1.0 to -8.3 kJ mol^-1 ^O_2_; Table 2) and purified Hbs (-7.6 to -13.7 kJ mol^-1^; Table 1) of coast and eastern moles, respectively, are notably lower than those reported for both whole blood (-16.8 and -14.4 kJ mol^-1^, respectively [37]) and the Hb fractions (-14.0 and -15.0 kJ mol^-1^, respectively [9]) of 'cold-adapted' musk-ox and reindeer. Given that possession of blood with numerically low oxygenation enthalpies helps to ensure adequate O_2 _delivery to cool peripheral tissues of these Arctic mammals [37], and that subterranean environments are generally moderate and thermally buffered from climatic extremes, what might account for the occurrence of this characteristic in the fossorial species (Figure 3, Table 2) but not in the (northerly distributed) semi-aquatic star-nosed mole? By mandating that blood-O_2 _affinity decreases as temperature increases, the exothermic character of the Hb oxygenation reaction also dictates that O_2 _uptake is compromised at high temperature. Unlike most fossorial mammals, talpid moles are powerful forelimb diggers that possess a large muscle mass surrounding the thoracic cavity. Consequently, we propose that the negligible thermal sensitivity of mole blood may minimize impairment of O_2 _loading at the lungs during exercise-induced hyperthermia while burrowing in hypoxic/hypercapnic soils. In fact, a high Δ*H *(whereby oxygenation is strongly exothermic) would further exacerbate O_2 _uptake potential since it increases the heat liberated upon oxygenation in the lungs (which accounts for up to 9% of metabolic heat production assuming a Δ*H *value of -42 kJ mol^-1 ^O_2 _[38]). The precise mechanism underlying the extremely low oxygenation enthalpy phenotype of coast/eastern mole blood is not known, but may be associated with the presence of an 'additional' Cl^- ^binding site in these species (see above).

The negligible effect of DPG on eastern mole Hb (Table 1), together with the finding that the erythrocytes of this species are nearly devoid of DPG (Table 3), demonstrates that this organophosphate does not have a central allosteric role in altering its blood O_2 _affinity. This reduced plasticity may be expected to have potentially adverse consequences since mammals with DPG-insensitive Hbs are unable to modify their Hb-O_2 _binding attributes in response to chronic changes in oxygen availability, and hence "are likely to be more restricted physiologically" than those whose oxygen affinity can be altered by increasing/decreasing red cell DPG concentrations [39]. Indeed, it has long been appreciated that cats, cows and sheep - which were subsequently discovered to possess low affinity, DPG-insensitive Hbs [16] - do not adjust well to high altitudes (hypoxia) [40]. Why then have eastern moles, following an extensive period of fossorial evolution (see Figure 1), recently forsaken the potential for adaptive modulation of their O_2_-binding affinity by phosphates [see e.g. [41]] and adopted a lower whole blood O_2 _affinity phenotype?

We hypothesize that the loss of DPG binding sharply increases the carrying capacity of eastern mole Hb for the metabolic end product CO_2_, thus providing a strong selective advantage for this subterranean inhabitant. Unlike carbon monoxide, CO_2 _does not bind to the heme iron, but instead can interact with the uncharged α-amino termini of the four globin chains to form carbamino CO_2_^- ^[42,43]. In the absence of DPG (which competes for this same binding site; see below), 70-80% of all carbamate formation under physiological conditions occurs on the "high affinity" β-type subunits [23,27]. In human Hb A, the carbamated β-chain N-termini are able to form intra-chain ionic contacts with β82Lys [43], thus stabilizing the deoxy state molecule (lowering Hb-O_2 _affinity). Conversely, the δ136Gly→Glu replacement in eastern mole Hb (through its neutralization of the cationic charge of δ82Lys) should disallow this δ1Val-δ82Lys interaction. Indeed, in the absence of DPG, the CO_2 _effect (ΔP_50_/Δ[CO_2_]) of the Hb Hope variant is 70% lower at a PCO_2 _of 40 mm Hg [23] than in human Hb A, and nearly 90% lower at a PCO_2 _of 80 mm Hg [21].

Within the erythrocytes of systemic capillaries, CO_2 _is largely hydrated to HCO_3_^- ^and H^+ ^by carbonic anhydrase. HCO_3_^- ^is subsequently exchanged with Cl^- ^via the band 3 protein anion-exchanger, and transported in the plasma [44]. However, band 3 protein anion-exchange appears to be the rate-limiting step in the uptake and offloading of CO_2_, potentially leading to exchange disequilibra in arteries and veins during severe exercise [44]. In this respect, the δ136Gly→Glu replacement in the eastern mole Hb may be significant as it deletes binding sites for anions (i.e., Cl^-^, lactate and DPG) between δ1Val and δ82Lys, thus largely freeing the 'high CO_2 _affinity' δ-chain N-termini from competitive binding constraints. Accordingly, this should result in enhanced binding of CO_2 _to eastern mole Hb (two molecules per tetramer) while reducing reliance on the anion-exchanger channel, thus increasing the maximal CO_2 _carrying capacity of eastern mole blood. In this regard, it should be noted that deoxygenation-linked carbamate formation also liberates a proton from the N-terminal α-amino group, which might be expected to increase the Bohr effect of eastern mole Hb (favouring O_2 _offloading). However, the Hb of this species possesses an additional external histidyl residue (δ69His; Figure 4) relative to other moles that should mitigate this effect. Finally, by stabilizing the tense (deoxy) conformation of eastern mole Hb, the δ136Gly→Glu replacement also confers a marked reduction in their whole-blood O_2 _affinity (a 5-6 mm Hg increase in their P_50_) compared to coast (see Figure 3) and European moles [1,3,4]. This reduced blood affinity would facilitate the offloading of O_2 _at a relatively high PO_2_, leading to a large O_2 _gradient between the plasma and tissues during burst tunnelling activities. This shift may negatively impact the O_2 _saturation of eastern mole blood during periods of hypoxia, but is presumably mitigated by elevated blood hematocrit (56.4%) and Hb concentrations (19.2 g dL^-1^; Table 3) compared to coast (46.8% and 17.4 g dL^-1^; [45]), star-nosed (50.5% and 17.2 g dL^-1^; [45]), Townsend's (46.4% and 16.9 g dL^-1^; [46]) and European moles (48.7% and 17.4 g dL^-1^; [1]). Eastern moles thus present a novel category of Hbs that appear to be specifically engineered for burst activities in gas-exchange impeded burrows.

## Conclusions

The capacity of certain mammals to withstand low-oxygen environments is thought to largely reside in the enhanced binding affinity of their blood Hb for oxygen. Here we document that following an extensive period of subterranean evolution, the lineage leading to present day eastern moles adopted a low oxygen affinity, DPG-insensitive blood phenotype - providing the first demonstration of this phenomenon in any mammal chronically exposed to hypoxia (and only the fourth known origin of this phenotype among mammals). The primary molecular mechanism involves an amino acid substitution (δ136Gly→Glu) that forms a salt bridge with δ82Lys of the same chain, thus deleting key binding sites for allosteric effectors (DPG, Cl^- ^and lactate) between δ1Val-δ82Lys and markedly reducing competition for CO_2 _binding (carbamate formation) at the N-terminus. Accordingly, we suggest this unique Hb phenotype enhances CO_2 _carrying capacity during burst activity (tunnelling) in gas-exchange impeded burrows.

## Methods

### Blood/tissue collection and preparation

#### Hemoglobin

All study animals were captured and cared for in accordance with the principles and guidelines of the Canadian Council on Animal Care under the authorization of a University-approved animal research protocol (University of Manitoba Animal Use Protocol# F01-026). Blood samples (~2 ml) were collected from anaesthetized eastern (Nashville, Tennessee) and coast moles (Abbotsford, BC, Canada) and immediately stored at -80°C. Samples were subsequently thawed and diluted with 1 volume distilled water, 0.1 vol. 1 M HEPES buffer (pH~7.5), and centrifuged for 15 min at 14,000 RPM. Individual isoHb components were isolated by preparative isoelectric focusing in a 110-ml LKB column containing a 1% solution of CO-saturated ampholines (pH range 6.7**-**7.7; LKB, Sweden) and eluted 1-ml fractions were analyzed for absorption (540 nm) and pH. Pooled fractions of individual Hb components were dialysed for 24**-**36 h against three changes of CO-equilibrated dialysis buffer (0.01 M HEPES), concentrated by ultrafiltration and frozen at -80°C in small aliquots that were thawed individually on ice prior to analyses. Air equilibrated samples showed slight if any spectrophotometric evidence for oxidation (< 5%).

#### Whole blood

Blood samples (~2 ml) were obtained in heparinized syringes via cardiac puncture from a single anaesthetized star-nosed mole (Caddy Lake, MB, Canada) and from coast and eastern moles collected from the same areas, but in different years, as those described above. A small sub-sample (60 μl) of blood was immediately transferred to a pre-heated tube (36-37 °C) and pH determined using a IQ Scientific benchtop ISFET pH meter with PH16-SS stainless steel micro pH probe.

Forelimb, hindlimb and heart muscles from six additional eastern moles euthanized with an overdose of Isoflurane inhalant anaesthetic were immediately dissected, freeze-clamped in liquid N_2_, and stored at -70°C. Tissue Mb and buffering capacity were later determined following the methods of Reynafarje [47] and Castellini and Somero [48] as outlined in McIntyre et al. [45]. Plasma and intraerythrocytic pH of blood sub-samples were measured using a freeze-thaw technique similar to that outlined by Zeidler and Kim [49]. Blood hematocrit and Hb concentrations were determined in duplicate following the procedures outlined by McIntyre et al. [45]. Standards were prepared from lyophilized human Hb (Sigma Hemoglobin Standard number 525-18). Samples were diluted to obtain values within the standard curve, and the Hb concentrations calculated by multiplying the measured value by the percent dilution. Red cell DPG concentrations were determined spectrophotometrically from four eastern moles with Roche kit Catalog Number 148 334, while those of three additional coast moles were assayed with Sigma Chemical kit No. 665-PA. Eastern mole red cell counts were determined from 1:200 dilutions of blood in Hayem's solution using a Neubauer hemocytometer, and mean corpuscle volume, mean corpuscular Hb and mean corpuscular Hb concentration calculated [50]. Similar hematology parameters from a single coast mole were measured in a Sysmex™ Model NE-8000™ hematology analyzer. Mean values between species were compared using a Welch's t-test, which accounts for possible unequal variances between the two sample means.

### Oxygen binding measurements

#### Hemoglobin

Immediately before O_2 _equilibration determinations, appropriate volumes of water, 0.1 M HEPES buffer, and when applicable, standard KCl and 2,3-DPG solutions were added to aliquots of purified Hb components (final Hb_4 _concentration 0.05 mM). Oxygen equilibration data were measured in duplicate at 25 and 37°C via absorbance changes at 436 nm using a modified diffusion chamber technique [51]. Ultrathin layers of Hb solutions (3 μl) were equilibrated alternatively with pure (> 99.998%) N_2 _and O_2 _then subjected to stepwise mixes of N_2 _and air prepared with two Wösthoff pumps connected in series to ensure full equilibration at each step [51]. P_50 _and *n*_*50 *_values interpolated from Hill plots were calculated from at least 4 equilibration steps between 30 and 70% saturation for each trial. Following binding measurements, Cl^- ^concentration for each sample was assessed using a CMT19 chloride titrator (Radiometer, Copenhagen, Denmark), and pH measured in oxygenated sub-samples equilibrated to experimental temperatures (25 and 37°C) using a Radiometer BMS2 Mk2 Blood Micro system and PHM 64 Research pH meter. Stock solutions of DPG added to Hb samples were assayed using Sigma enzymatic test chemicals. The overall enthalpy of oxygenation (Δ*H*, kJ mol^-1 ^O_2_), corrected for the solubilization heat of O_2 _(-12.5 kJ mol^-1^), was calculated from the integrated van't Hoff equation [9].

#### Whole blood

Blood-oxygen binding properties were measured by equilibrating small (3-4 μl) aliquots of whole blood to O_2 _tensions ranging from 0 to 190 mm Hg using a modified [52] Hem-O-Scan (American Instrument Co, Silver Springs, MD). Initially, measurements were conducted at the typical body temperature (T_b_) of each species (Eastern mole = 36.0°C; coast mole = 36.0°C, KLC, unpublished data; star-nosed mole = 37.7°C, [53]) at a PCO_2 _of 38 mmHg. This procedure was repeated in CO_2_-free gas, and the pH of separate deoxygenated blood sub-samples equilibrated (at T_b_) to these PCO_2_'s were used to calculate CO_2 _Bohr coefficients. To assess the effect of temperature on oxygen equilibration curves, trials were also conducted at 32 and 40°C for each species. Because star-nosed moles are semi-aquatic and may exhibit strong regional heterothermy while foraging in cold water, additional trials on blood samples equilibrated to 3, 15 and 36°C were conducted. Oxygen equilibration curves were constructed following Severinghaus [54].

### DNA/RNA extraction and cDNA library construction

Genomic DNA was prepared from 100-200 mg of spleen or liver tissue using standard phenol/chloroform extraction procedures. Primers were designed using areas of high sequence identity in the coding, and 5' and 3' flanking regions of orthologous eutherian α- and β-like globin genes and from the published amino acid sequences for these polypeptide chains of the European mole [5]. To determine appropriate annealing temperatures for each primer pair (see Additional file 1: Table S1), PCR reactions were initially run on 100 ng of template DNA and *Taq *polymerase using the gradient function of a MJ Research Dyad™ thermal cycler. Following a 5 min denaturation period at 94°C, a standard three-step PCR protocol was used (94°C for 30 sec; 48-56°C for 15 sec; 72°C for 60 sec; 30 cycles). The 5' and 3' flanking regions of each gene were subsequently obtained using the APAgene™ Genome Walking Kit (Bio S&T Inc., Montreal, PQ). In all cases, amplified PCR products of the desired size range were excised from the 1% agarose gel and purified using the Qiagen MinElute Gel Extraction Kit. These products were then cloned into Qiagen pDrive cloning vectors and positive clone plasmids purified with the Qiagen QIAprep Spin Miniprep Kit.

Total RNA was extracted from ~80 mg of eastern (*n *= 3) and coast mole (*n *= 3) spleen samples that had been stored in RNAlater (Ambion) using TRIzol^® ^reagent, as per the manufactures directions (Invitrogen). The quantity and quality of the recovered RNA was determined using an Ultrospec™ 3100 *pro *UV/Visible Spectrophotometer (Amersham Biosciences). 10 μg of total RNA was used to construct a cDNA library using an ExactStart™ Full-Length cDNA Library Cloning Kit (Epicentre Biotechnologies). RNA decapping, 5' oligo ligation and first strand cDNA synthesis reactions were performed according to Epicentre Biotechnologies' protocol. Second strand cDNA was synthesized and amplified by a PCR reaction containing a 2 μl aliquot of first strand cDNA and 48 μl of reaction mix (0.25 μl of each dNTP (2.5 mM), 5 μl of 10 × Reaction Buffer (Invitrogen), 1.5 μl of 50 mM MgCl_2_, 1 μl of each primer (provided with the ExactStart™ Kit), 0.4 μl (2.5 Units) of Taq DNA polymerase (Invitrogen) and 38.1 μl of ddH_2_O), using an Eppendorf Mastercycler^® ^Gradient thermocycler. Following an initial denaturation period of 95°C for 30 seconds, total cDNA was amplified using a 3-step PCR protocol (95°C for 30 seconds; 60°C for 30 seconds; 72°C for 4 minutes; 20 cycles). The double stranded cDNA was purified by phenol:chloroform extraction then digested with *Asc I *and *Not I *restriction enzymes and ligated into pCDC1-K cloning ready vectors according Epicentre Biotechnologies' protocol. 1 μl of the ligation reaction was used to transform 50 μl of TransforMax™ EC100™ Chemically Competent *E. coli *(Epicentre Biotechnologies). The transformation reaction was incubated at 37°C with shaking (225 rpm) for 1 h to allow the expression of the kanamycin resistance gene. In order to establish a cDNA library culture, the entire transformation reaction was used to inoculate Luria Bertani broth (10 ml final volume) containing kanamycin (50 μg ml^-1 ^final concentration) and incubated at 37°C with shaking (225 rpm) for 18 h. A 3 ml aliquot of this culture was purified using a QIAprep^® ^Spin Miniprep Kit to obtain a pure plasmid cDNA library.

### Clone selection

Positive clones were selectively retrieved from the plasmid library using a modified version of the magnetic bead cDNA capture method described by Shepard and Rae [55]. In brief, the plasmid cDNA library was hybridized with biotinylated oligonucleotide probes (which target highly conserved regions of the α- and β-like globin genes) and blocking oligonucleotides (which correspond to the 5' and 3' ends of each probe to prevent renaturation of the plasmid DNA; Additional file 2: Table S2). Plasmids that hybridized with a biotinylated probe were bound to streptavidin coated magnetic beads (Dynabeads^® ^M-280 Streptavidin, Invitrogen) and then subjected to a series of high stringency washes to remove any plasmids that non-specifically hybridized with a probe. The remaining plasmids were released from the magnetic beads and transformed into TransforMax™ EC100™ Chemically Competent *E. coli *(Epicentre Biotechnologies). Transformed cells were spread on plates containing 50 ml of LB agar, 50 μl of Kanamycin (50 μg μl^-1^), 80 μl of X-gal (40 mg ml^-1^) and 20 μl of IPTG (0.1 M) and incubated at 37°C for 18 h.

Selected clones were screened for the presence of their respective insert by scraping an isolated colony into a 0.2 ml PCR tube and adding 15 μl of a PCR reaction mix (0.35 μl of each dNTP (2.5 mM), 1.5 μl of 10 × Reaction Buffer (Invitrogen), 0.6 μl of 50 mM MgCl_2_, 0.6 μl of each primer (designed from highly conserved regions among mammalian α- and β-globin genes using Primer Premier 5.0 software), 0.12 μl (0.6 Units) of Taq DNA polymerase (Invitrogen) and 10.18 μl of ddH_2_O). Positive clones were used to inoculate 8 ml of LB culture medium containing kanamycin (50 μg ml^-1 ^final concentration) and incubated for 18 h at 37°C while shaking at 225 rpm. A 3 ml sample of each culture was purified using a QIAprep^® ^Spin Miniprep Kit (Qiagen). 2 μl of purified plasmid DNA was digested with Eco RI and Hind III (1 μl of each enzyme, 2 μl of 10 × React^®^2 buffer and 14 μl of ddH_2_O) and electrophoresed for 1 hr at 100 V on a 1% agarose gel (UltraPure™, Invitrogen) to confirm the size of the insert.

### DNA sequencing

Sequencing reactions were preformed on 200 ng of purified plasmid DNA using the BigDye^® ^Terminator v3.1 Cycle Sequencing Kit (Applied Biosystems) and the universal sequencing primer M-13(R). Reaction mixtures were sequenced using a 4-capillary Applied Biosystems 3130 Genetic Analyzer. Consensus alignments for each gene were constructed using Sequencher™ (Version 4.6) software, and the amino acid compliment of each globin chain was deduced. Sequence data were deposited in GenBank with the accession numbers (AY842447-AY842448, HM060229-HM060234 and HM060237-HM060243).

### Determination of Hb isoform composition

After using isoelectric focusing native gels to assess Hb isoform diversity, the individual globin chain subunits were dissociated and separated by means of Acetic acid-Urea-Triton X-100 (AUT) gel electrophoresis [56]. Electrophoretic bands representing dissociated α- and β-type chain monomers were excised from each AUT gel, digested with trypsin, and identified by means of tandem mass spectrometry (MS/MS; [57,58]). The peak lists of the MS/MS data were generated by Distiller (Matrix Science, London, UK) using the charge state recognition and de-isotoping with default parameters for quadrupole time-of-flight data. Database searches of the resultant MS/MS spectra were performed using Mascot (Matrix Science, v1.9.0, London, UK). Specifically, the peptide mass fingerprints were used to query a reference database of α- and β-like globin sequences that included each of the globin cDNA sequences from the same sample of moles. The following search parameters were used: no restriction on protein molecular weight or isoelectric point, enzymatic specificity set to trypsin, and methionine oxidation allowed as a variable peptide modification. Mass accuracy settings were 0.15 daltons for peptide mass and 0.12 daltons for fragment ion masses. We identified all significant protein hits that matched more than one peptide with *P *< 0.05.

### Phylogenetic reconstructions

To infer orthologous relationships of β-like globin sequences from eastern mole and coast mole, we conducted a phylogenetic survey of nucleotide variation in the β-like globin genes of six other eutherian mammals. In addition to eastern mole and coast mole, this set of species included three other eulipotyphlan species (*Atelerix albiventris *[GenBank: AC104389]; *Erinaceus europaeus *[scaffolds 283493, 67442, and 340990]; and *Sorex araneus *[AC166888]), as well as human (*Homo sapiens *[AC104389]), rabbit (*Oryctolgus cuniculus *[AC166202]), and armadillo (*Dasypus novemcinctus *[AC151518]). β-like globin sequences from human, rabbit, and armadillo were included in the phylogenetic analysis because they each possess a closely linked pair of well-characterized *HBB *and *HBD *genes [13]. Sequences were aligned using MUSCLE [59], as implemented in the European Bioinformatics Institute web server http://www.ebi.ac.uk. Since the coding regions of duplicated globin genes are often affected by gene conversion, reliable inferences about orthologous relationships can be obtained by examining intron 2 sequence [13,14]. We therefore performed phylogenetic reconstructions based on both coding sequence (477 bp) and intron 2 sequence (938 bp). We inferred phylogenetic relationships among the β-like globin sequences in a maximum likelihood framework using Treefinder, version April 2008 [60], and we assessed support for each node with 1000 bootstrap pseudoreplicates. We selected the best-fitting model of nucleotide substitution for each of the two data partitions using the Bayesian Information Criterion in Treefinder. Phylogenetic reconstructions of the coding and intronic sequences were conducted using the HKY + γ and TN + γ models, respectively.

### Molecular modeling

Amino-acid substitutions of both mole species (relative to human Hb A) were inserted into the 3D T-state deoxy structures of human Hb with DPG absent (eastern mole model from PDB 2DN2) and present (coast mole model from PDB 1B86). Structural homology models of eastern mole and coast mole Hbs were then prepared using the MODELLER function of the Insight II program package version 97.2 (Biosym Technologies, San Diego, CA). The strain energy in the vicinity of the central cavity between the δ-chains of both Hb models were generated separately in the GROMOS force field using the 53A6 parameter set optimized for molecular dynamics simulations [61]. For each substitution the strain energy was subsequently minimized using the GROMACS package (version 3.3). This involved a brief steepest descents run that employed a maximum step size protocol of 1Å, and a maximum tolerance of 1000 kJ mol^-1 ^nm^-1^. This was followed by a more extensive conjugate gradients minimization with a tolerance of 100 kJ mol^-1 ^nm^-1^. A Morse oscillator model was used to represent covalent bonding in the conjugate gradients minimization step, while a harmonic oscillator approximation was utilized for the steepest descents protocol. For the eastern mole modelling, the N-terminus was initially set to a charge of +0.5. Under these conditions, two equally likely intra-chain electrostatic interactions were present: Gluδ136-Lysδ82 and Gluδ136-Valδ1 (ionized N-terminus). However, given that the former bond is much more stable than the latter, the equilibrium shifts solely to the Gluδ136-Lysδ82 formation over multiple iterations. This same association is exclusively found when the N-terminus was given a neutral charge. Three-dimensional molecular representations were visualized with DINO version 0.9.1 [62].

## Authors' contributions

KLC and REW designed research; KLC, JFS, JS, AVS, AP and JB performed research; KLC, JS, JFS, HM, KC, JB and REW contributed new reagents/analytic tools; KLC, JS, AVS, JFS and HM analyzed data; KLC drafted the manuscript; and KLC, JFS and REW contributed to the final manuscript writing and its revisions. All authors have read and approved the final manuscript

## Supplementary Material

Additional file 1 **Table S1**. Primers used to amplify the *HBA *and *HBD *genes from coast and eastern mole DNA. Click here for file

Additional file 2 **Table S2**. Oligonucleotides used to probe the *HBA *and *HBD *genes from coast and eastern mole cDNA. Click here for file
