# Supplementary material for: Molecular basis of a novel adaptation to hypoxic-hypercapnia in a strictly fossorial mole
Source: BMC Evol Biol. 2010 Jul 16;10:214. doi: 10.1186/1471-2148-10-214 (PMC2927915; doi:10.1186/1471-2148-10-214)
Supplement: Additional file 1 — Table S1. Primers used to amplify the HBA and HBD genes from coast and eastern mole DNA. [file 1471-2148-10-214-S1.DOC]

**Additional file – Campbell et al**

**Supplemental Table 1** – Primers used to amplify the *HBA* and *HBD* genes from coast and eastern mole DNA.

| **Oligonucleotide** | **Sequence (5`→3`)** |
| --- | --- |
| ***HBA* primers** |  |
| HBA 1F | GCACCGACAAGACCAACATC |
| HBA 2R | GGTACTTGGAGGTCAGCACG |
|  |  |
| HBA walk R1 | AAGTTGACGGGGTCCACACGCAGC |
| HBA walk R2 | TTGTGCGCGTGCAGGTCGCTCA |
| HBA walk R3 | TTGCCGTGGGCTTTGACCTGGG |
|  |  |
| ***HBD* primers** |  |
| HBD 1F | GTGGGGCAAGGTGAATGTGG |
| HBD 2R | CATCACACACACCAGCACG |
|  |  |
| HBD walk F1 | AAGGAAGTATCTAGTGATGGAAGAGCAG |
| HBD walk F2 | AAGAGCAGAAGTAGAAAAGAAGGAAGCAG |
| HBD walk F3 | CAACCCATGGTCACAATTAACATATATGC |
|  |  |
| HBD walk R1 | CAGCTCACTCAGCTTGGCATAGGTGC |
| HBD walk R2 | CCTTCACCTTAGCATTGCCCATGATAGC |
| HBD walk R3 | AAAGAACCTCTGGGTCCAGGGGTAGAC |
