# Supplementary material for: Molecular basis of a novel adaptation to hypoxic-hypercapnia in a strictly fossorial mole
Source: BMC Evol Biol. 2010 Jul 16;10:214. doi: 10.1186/1471-2148-10-214 (PMC2927915; doi:10.1186/1471-2148-10-214)
Supplement: Additional file 2 — Table S2. Oligonucleotides used to probe the HBA and HBD genes from coast and eastern mole cDNA. [file 1471-2148-10-214-S2.DOC]

**Additional file – Campbell et al**

**Supplemental Table 2** – Oligonucleotides used to probe the *HBA* and *HBD* genes from coast and eastern mole cDNA.

| **Oligonucleotide** | **Sequence (5`→3`)** |
| --- | --- |
|  |  |
| HBA biotin probe | biotinCCACCACCAAGACCTACTTCCCCCA |
| HBA 5’ block | TACGGCGGGGAGGCCCTGGAGAGGATGTTCGACTGCTTCC |
| HBA 3’ block | CTTCGACATGAGCCCCGGGTCCGCCCAGGTCAAAGCCCAC |
|  |  |
| HBD biotin probe | biotinGAGGTTCTTTGACAGCTTYGGTGACCT |
| HBD 5’ block | GGCCCTGGGCAGGCTGCTGGTTGTCTACCCCTGGACCCA |
| HBD 3’ block | GTCCTCTCCTGCTGCTATCATGGGCAATCCTAAGGTGAA |
